# Role of Fc in Antibody-Mediated Protection from Ricin Toxin

**DOI:** 10.3390/toxins6051512

**Published:** 2014-05-07

**Authors:** Seth. H. Pincus, Anushka Das, Kejing Song, Grace A. Maresh, Miriam Corti, Jody Berry

**Affiliations:** 1 Children’s Hospital New Orleans, New Orleans, LA 70118, USA; E-Mails: anushkadas.11@gmail.com (A.D.); ksong2@lsuhsc.edu (K.S.); gmaresh10@gmail.com (G.A.M.); miriam.corti@cox.net (M.C.); 2 Department of Pediatrics and Microbiology, Louisiana State University Health Sciences Center, New Orleans, LA 70112, USA; 3 Tulane University, New Orleans, LA 70118, USA; 4 Cangene Corporation (now Emergent Biosolutions Inc), Winnipeg, MB R3T 5Y3, Canada; E-Mail: jody_berry@bd.com

**Keywords:** ricin, monoclonal antibody, polyclonal antiserum, Fc region, Fc receptor, Fab, neutralization, *in vivo* protection

## Abstract

We have studied the role of the antibody (Ab) Fc region in mediating protection from ricin toxicity. We compared the *in vitro* and *in vivo* effects of intact Ig and of Fab fragments derived from two different neutralizing Ab preparations, one monoclonal, the other polyclonal. Consistent results were obtained from each, showing little difference between Ig and Fab in terms of antigen binding and *in vitro* neutralization, but with relatively large differences in protection of animals. We also studied whether importing Ab into the cell by Fc receptors enhanced the intracellular neutralization of ricin toxin. We found that the imported Ab was found in the ER and Golgi, a compartment traversed by ricin, as it traffics through the cell, but intracellular Ab did not contribute to the neutralization of ricin. These results indicate that the Fc region of antibody is important for *in vivo* protection, although the mechanism of enhanced protection by intact Ig does not appear to operate at the single cell level. When using xenogeneic antibodies, the diminished immunogenicity of Fab/F(ab’)_2_ preparations should be balanced against possible loss of protective efficacy.

## 1. Introduction

It is clear that neutralization of toxins by Ab plays a major role in protective immunity. Critical vaccines (e.g., DPT) and passive Ab therapies are based upon this fact, which represents one of the few generally-agreed upon “truths” in the field of human vaccinology. Yet how exactly Abs protect us from toxins is not fully understood. We generally teach our students that Ab functions by preventing attachment and internalization of the toxin to target cells [1,2], suggesting that anti-B chain immunity would be paramount. But so many exceptions to this generalization have been described, including ricin [3,4], that toxin neutralization likely involves multiple mechanisms, some unique to the individual toxin and its mode of pathogenicity [5]. For example, we have shown that the most protective Abs target ricin-A chain, and that neutralization occurs inside the cell [4], as others have demonstrated for shiga toxin, ricin’s cousin [6].

The role of the Fc region of Ab in protective efficacy is also not fully defined. As a generalization, toxin neutralization has primarily been considered due to Ab binding the toxin and blocking its activity, a V-region function [1,2]. Consequently xenogeneic Abs used in passive immunotherapy are frequently prepared in the form of Fab/F(ab’)_2_ fragments, with the intent to limit immunogenicity and risk of “serum sickness” in such preparations [5,7,8]. However, recent work, using Fc-receptor (FcR) knockout mice, suggests that FcR function is a “requirement” for protection against anthrax toxin [9,10]. In this manuscript, we further examine this apparent paradox, employing ricin-neutralizing Abs to study the role of Fc-mediated protection. Both monoclonal (mAbs) and polyclonal (pAbs) were used to evaluate protection of individual cells and in mice. Further, we asked what effect would the transport of protective Abs into the cells by FcRγ have upon intracellular neutralization of ricin toxicity [4].

## 2. Results and Discussion

### 2.1. Comparison of Intact IgG and Fab/F(ab’)_2_ for Binding, Neutralization, and in vivo Protection

**Figure 1:**
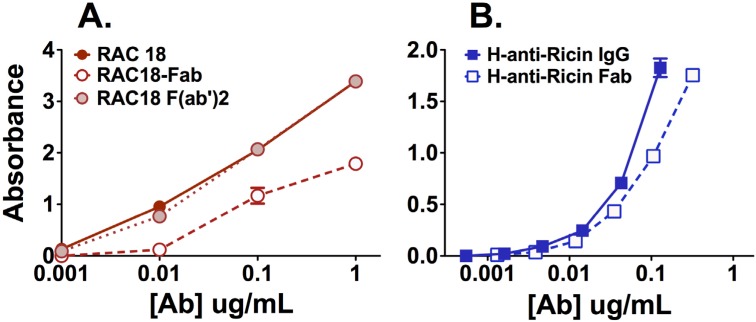
Comparative binding of intact Ig and Fab fragments to ricin toxin. Two different anti-ricin Abs were titrated by ELISA for binding to ricin. Results are the mean and SEM of triplicate samples. (**A**) Murine mAb RAC18 (solid line) and the corresponding Fab (dashed line) and F(ab’)_2_ (dotted line) fragments; (**B**) Polyclonal horse-anti-ricin igG (solid line) and corresponding Fab/F(ab’)_2_ fragments (dashed line).

To study the role of Fc-mediated effects on ricin neutralization, we compared the function of intact IgG and Fab/F(ab’)_2_ fragments using two very different Ab preparations. The first, RAC18, is a mAb that neutralizes equivalently in murine or chimeric (murine-V, human-gamma-1/kappa) versions. This Ab binds at the A-chain enzyme active site, blocking its N-glycosidase function, effectively blocks ricin cytotoxicity in tissue culture, and is highly protective *in vivo*. Both Fab and F(ab’)_2_ fragments were made from RAC18, a murine IgG2a/κ, although F(ab’)_2_ fragments were only used in ELISA (Figure 1) The other preparation we studied was a polyclonal horse Ab that binds to both ricin A and B chains. Very large quantities of this pAb have been produced. A “despeciated” preparation of this Ab consists of a mixture of Fab/F(ab’)_2_ fragments, primarily the latter [5]. We then compared intact IgG and fragments from the two sets of Abs for binding to ricin by ELISA, neutralization of ricin cytotoxicity *in vitro*, and protective efficacy *in vivo*.

#### 2.1.1. Fab Fragments Bind Ricin Less Avidly than Intact Ab or F(ab’)_2_

ELISA was performed to measure binding to ricin by two anti-ricin Abs, RAC18 and horse-anti-ricin, and their corresponding Fab/F(ab’)_2_ fragments. Binding of murine anti-ricin was detected with specific anti-kappa chain Ab, which binds intact Ig or fragment equivalently. Detection of the horse pAb was with an anti-H+L chain secondary, which could have biased the results against fragments. Results (Figure 1) show that intact IgG binds most avidly for both RAC18 and horse-anti-ricin. For RAC18, binding of intact IgG and F(ab’)_2_ is equivalent, whereas the binding of the Fab fragment is markedly lower. These results suggest that valency is important for ELISA binding. For RAC18, both bivalent preparations clearly outperformed the monovalent Fab. The horse pAb fragments are a mixture, but mostly bivalent fragments, and binding was only minimally decreased, although we cannot rule out that had we tested higher concentrations of Ab and Fab, greater differences may have been observed.

#### 2.1.2. Fab Fragments and Intact IgG Neutralize Ricin Cytotoxicity Equally

**Figure 2:**
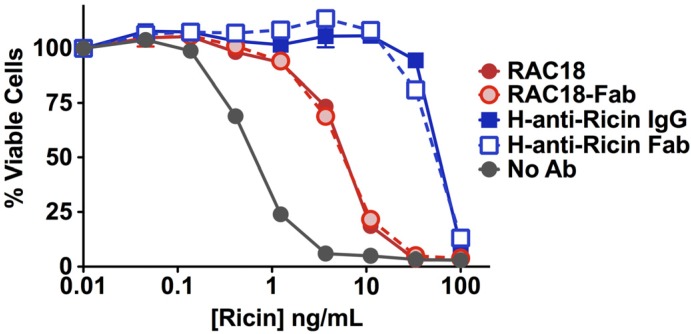
Neutralization of ricin toxicity by intact Ab or Fab fragments. Comparative molar concentrations of ntact IgG (1 µg/mL) or fragment (0.66 µg/mL) were mixed with the indicated concentration of ricin. H9 cells (2.5 × 10^4^ per well) were added. Two days later, cell viability was measured by MTS dye reduction. Data are mean and SEM of triplicate determinations.

The ability of Ab or fragment to neutralize ricin cytotoxicity was measured with an MTS dye-reduction assay, which assays oxidative metabolism as an indicator of cell viability (Figure 2). Two observations arise from the data. First, horse pAb was 10×–30× more effective at neutralizing ricin than murine mAb. This may reflect either higher avidity binding or an additive effect resulting from binding to multiple epitopes. The second observation is that there was no difference in the ability of intact IgG or Fab to neutralize ricin. In light of the marked difference in binding of RAC18 IgG and Fab to ricin, as measured by ELISA, the observation that both neutralized equally was unexpected.

#### 2.1.3. Antibody Fc is Important for *in vivo* Protection

**Figure 3:**
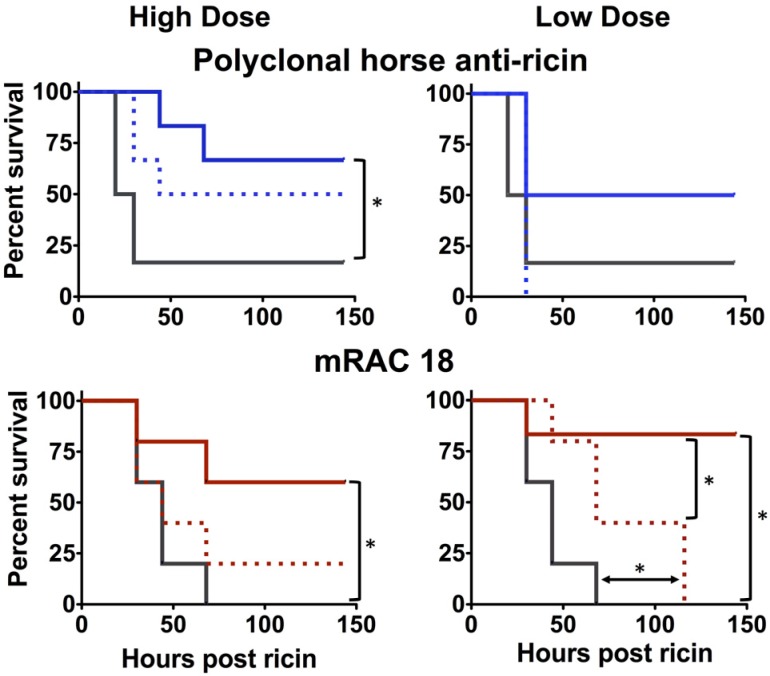
Protective efficacy of intact IgG *vs.* Fab/F(ab)’_2_ fragments. Mice received intact Ab (high dose: 3 mg/kg, low dose: 1 mg/kg) or Fab (mouse), or Fab/F(ab)’2 (horse) (high dose: 2 mg/kg, low: 0.66 mg/kg) four hours after a ricin challenge (20 µg/kg). Survival post-challenge is shown. Intact antibody is shown with a colored solid line, Fab a dotted line. Control animals are shown in black. Significance by Mantel-Cox log rank test (*p* < 0.05) is indicated with an asterisk.

We have utilized a well-characterized murine model to study the ability of Ab to protect against parenteral injection of ricin toxin [3,11]. To increase the stringency of the challenge, Ab was administered 4 h after ricin. This mimics part of the delay that would occur in a human exposure. If we delay any longer, the animals would not be salvageable by any antibody, and we could not compare our preparations. In the first experiment, mice were challenged with ricin and then given high or low dosages of either intact IgG or Fab fragments (Figure 3).

The intact IgG provided significant protection in all cases, except low dose horse pAb. Fab fragments provided less protection than intact IgG, and in only one example (low dose RAC18) was this significantly better than no Ab. In the experiment testing the murine mAbs and Fab fragments, survival of the untreated control animals was less than that observed in the experiment comparing the polyclonal Abs. Thus the requirements for protection by the murine mAbs may have been more stringent.

**Figure 4:**
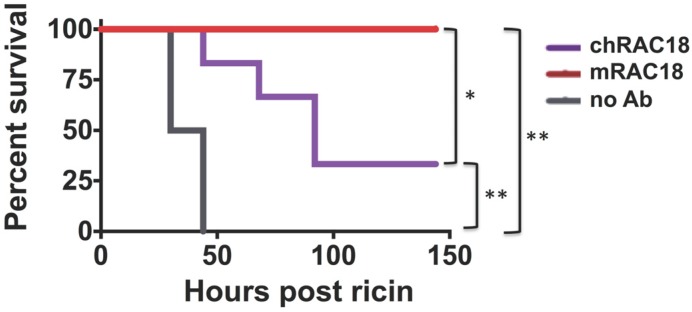
*In vivo* protection from ricin toxicity by murine or chimeric RAC18. Mice received 1 mg/kg murine or chimeric mAb 4 h after ricin challenge. Survival post-challenge is shown. Statistical significance is displayed: * *p* = 0.05; ** *p* = 0.01.

A second *in vivo* experiment demonstrated that murine RAC18 protected better than a chimeric mouse/human RAC18, showing that mouse Fc regions perform better in mice than human Fc’s (Figure 4). These results indicate that whereas Fc region does not appear to play a role in Ab binding or *in vitro* neutralization, the Fc does play a role in protecting an intact animal, the only parameter of any practical importance. The results also show that the superiority of the horse pAb over RAC18 for *in vitro* neutralization was not maintained *in vivo*.

### 2.2. Fc Receptors Do Not Affect Ab-Mediated Protection from Ricin Cytoxicity

In Section 2.1, we showed a discrepancy regarding the role of the Fc region on Ab-mediated protection from toxin. Fc seems irrelevant for cytoprotection (Figure 2), but rather important for *in vivo* protection (Figure 3 and Figure 4). One potential explanation for this difference is the potential role of FcRγs on Ab function in an intact animal. While there are a variety of Fc functions mediated by other factors (e.g., complement), FcRγs I–III mediate important effector functions, and immune regulation. Moreover, others have defined a requirement for FcR function in protection from the effects of anthrax toxin [10]. To examine the role of IgG-binding FcRs in an experimentally simple system, we made use of chimeric RAC18 and a set of cell lines that either didn’t express FcR, or were transfected to express human FcRγs I, IIa, IIb, or IIIa [12]. We have previously shown that RAC18 predominantly neutralizes ricin inside the cell [4], suggesting that transport of IgG into the cell by Fc receptors might enhance neutralization. Here we show that RAC18 binds to FcRs and can be transported into the cell. We then address the following questions: where does Ab preloaded into the cell via FcR-mediated internalization localize, does ricin cross paths with Ab as it is internalized, and does internal Ab neutralize ricin? To perform such studies, it is necessary for the Ab to have achieved a steady state concentration within the cell, before it is then washed away and ricin added. The internalized mAb localizes in compartments to which ricin traffics only at later time points, and offers no evidence of enhanced protection. Any protective advantage may be offset by increased internalization of ricin when RAC18, FcRγ, and ricin are all present concurrently.

#### 2.2.1. Chimeric RAC18 is Bound by FcRI and FcRIIb Expressed on the Cell Surface

**Figure 5:**
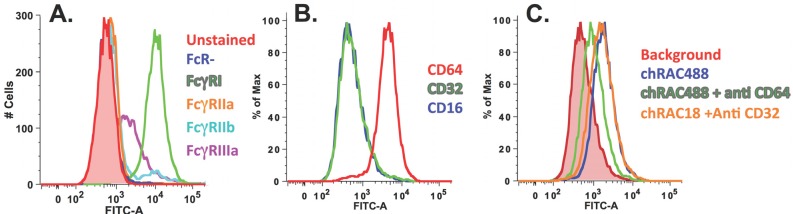
Chimeric RAC18 is bound by human FcRI. (**A**) FcRγ expressing cells were incubated with Alexa-488 conjugated chRAC18 (5 µg/mL) for 1 h in PBS/BSA/Azide, washed, and then analyzed by flow cytometry; (**B)** FcRγI cells express CD64 but not CD16 or CD32. Cells were stained with primary Ab at 0.25–0.5 µg/mL and then FITC anti-mouse IgG secondary Ab; **(C**) Binding of chimeric RAC18 to FcRγI cells is only inhibited by anti-CD64, not anti-CD32. Cells were first incubated with anti-CD32 or CD64 (0.25–0.5 µg/mL) and then with Alexa-RAC18 (1 µg/mL).

As a first step, we determined whether chRAC18 bound to FcRγs on the surface of transfected HeLa cells. Unfixed cells were incubated with Alexa-488 conjugated chRAC18. No ricin was present. Cells were washed and studied by flow cytometry (Figure 5). The results showed strong binding by cells expressing FcRγI, with less binding to FcRγIII, and no binding to the other FcRγs (Figure 5A). We then confirmed that the cells purported to express FcRγI, did indeed express this receptor, as demonstrated by the binding of anti-CD64 mAb, but not anti-CD16 or anti-CD32. We showed that the chRAC18 was bound via FcRγI by blocking the binding with anti-CD64 mAb, but not anti-CD32.

#### 2.2.2. Localization of RAC18 Internalized by FcRγI

**Figure 6:**
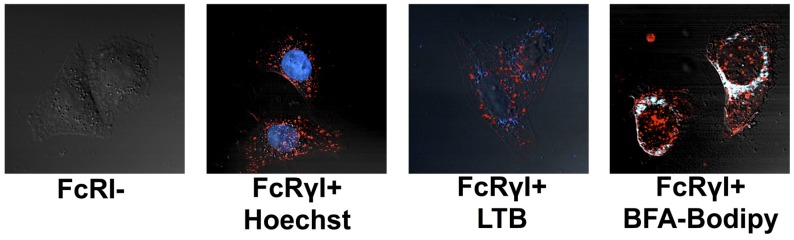
Localization of chRAC18 internalized by FcRγI. Cells were incubated with 10 µg/mL of Alexa-labeled chRAC18 (**red**) for 18 h. For the final 30–60 min, cells were incubated with Bodipy-BFA (250 ng/mL), Hoechst dye (2 µg/mL), or Lysotracker Blue (LTB, 125 nM), all shown in blue. Colocalization of dye and Ab is shown in white.

To determine the intracellular localization of Ab after it has been internalized by FCRγI, cells adherent to optical petri dishes were incubated with the Alexa-conjugated chRAC18 for 18 h under tissue culture conditions. The cells were washed, organelle specific dyes added, cells incubated for 30–60 min, and visualized by confocal microscopy (Figure 6). FcR− cells do not take up any chRAC18 (red), whereas the FcRγI+ cells do. The mAb does not colocalize with Hoechst dye or Lysotracker blue, indicating the mAb is extranuclear, and not in lysosomes. chRAC18 was found throughout the endoplasmic reticulum and Golgi, since almost all Bodipy-BFA colocalizes with mAb. However, mAb also accumulated in other compartments, not marked by bodipy-BFA.

#### 2.2.3. Colocalization of Ab Internalized by FcRγI with Ricin

**Figure 7:**
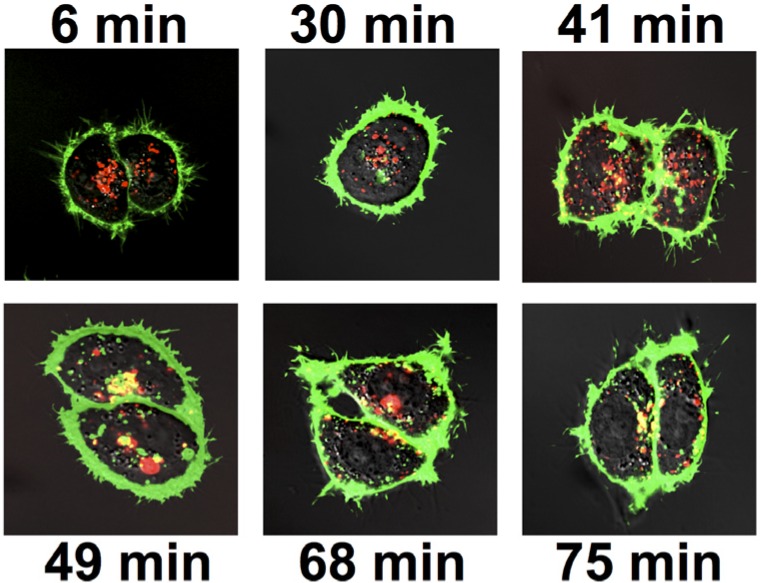
Colocalization of internalized chRAC18 with entering ricin in FcRγI cells. Cells were incubated with 10 µg/mL of Alexa-labeled chRAC18 (**red**) for 18 h, washed, placed on a heated stage and Alexa-labeled ricin (3 µg/mL, **green**) added. Different cells were visualized at 2–3 min intervals. Representative time points are shown. Colocalization of ricin and mAb appears orange/yellow.

In trafficking to its site of action, RAC traverses the endoplasmic reticulum and Golgi [4,13,14,15], where mAb internalized by FcRI localize. To study if Ab and ricin meet in the cell, we examined the colocalization of internalized mAb with ricin as it enters and traffics through the cell (Figure 7).

FcRγI cells were incubated with Alexa-conjugated chRAC18 (red) for 18 h. The cells were washed and visualized at times following the addition of labeled ricin (green). Colocalization is shown in orange. Ricin accumulated at the cell surface within min, and had begun to enter the cell by 30 min, although ricin did not colocalize with Ab until 41 min. Colocalization increased with time. But even at later time points, unbound ricin was present within the cell. We tested the ability of this internalized Ab to protect cells from ricin, by using an 18 h pre-incubation period with chRAC18, washing out extracellular mAb, adding varying concentrations of ricin, and measuring cell survival. There was no difference in survival between FcR− and FcRγI+ cells, nor between cells with and without intracellular RAC18 (data not shown).

#### 2.2.4. Effect of FcR on Internalization of Ricin and Neutralization by RAC18

**Figure 8:**
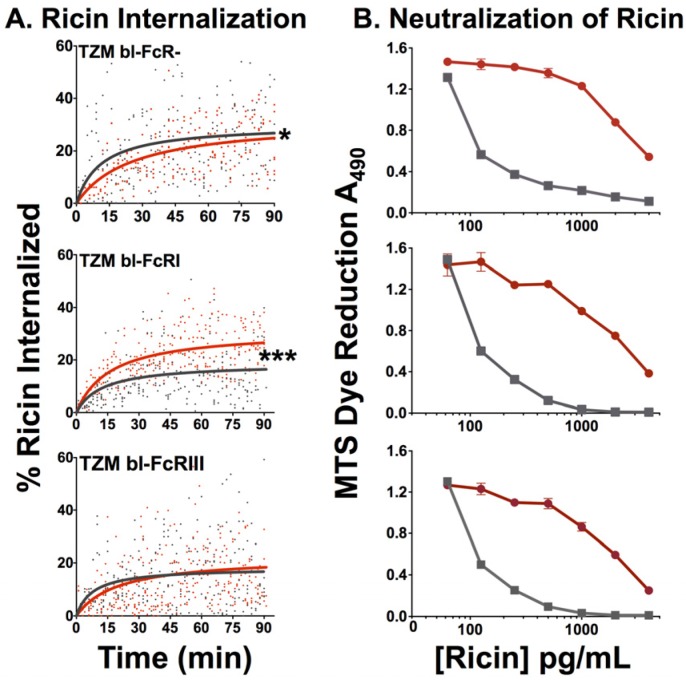
Effects of FcR on internalization of ricin and protection from ricin-mediated cytotoxicity. (**A**) Measurement of ricin internalization was performed by confocal microscopy over a 90 min period on a heated stage in the presence or absence of 30 µg/mL of chRAC18. The amount of internalized ricin was measured by manual partitioning and calculated from pixilated values obtained from the partitioning. Measurements performed in the presence of chRAC18 are shown in red, in the absence of mAb in black. The results are combined data from three independent experiments. Individual data points and the best fit curves are shown. Statistical significance was determined by the F test: * *p* < 0.05; *** *p* < 0.0001; (**B**) Neutralization by RAC18 was not affected by FcR expression on cells. MTS assay was performed in the presence (**red**) or absence (**black**) of chimeric RAC18 (20 µg/mL) at the indicated concentrations of ricin.

Passive internalization of opsonized antigen has been proposed as a mechanism of disease, for example the enhanced disease and hemorrhagic fever associated with a second dengue virus infection of a different serotype [16]. We asked whether this phenomenon may also occur with ricin toxin, *i.e.*, if more toxin enters FcRγ+ cells in the presence of Ab, than in its absence. We measured internalized ricin using quantitative confocal microscopy techniques that have been detailed and validated elsewhere [4]. Live cells were first exposed to chRAC18 and immediately afterwards Alexa-ricin was added without washing the cells, so that mAb and ricin were both present. Different cells were imaged every 2–3 min for 90 min. The percent ricin internalized was plotted against time (Figure 8A). The data were fitted with a nonlinear regression using the formula: y = M*x*/(T_1/2_ + *x*), where M is the maximal value and T_1/2_ is the time to reach the half maximal value. The results show that in cells lacking FcR, internalization of ricin is slowed by mAb. The difference is small, but statistically significant. The increased size of ricin-anti-ricin immune complexes seems a likely explanation. However, in cells bearing FcRγI, to which chRAC18 binds strongly, internalization of ricin is markedly increased in the presence of mAb, presumably via enhanced internalization of immune complexes by FcRγI. In cells with the weaker binding FcRIII, the two effects appear to counterbalance.

In Figure 8B, we examined whether the presence or absence of FcR affects the ability of chRAC18 to protect cells from ricin-mediated cytoxicity. MAb was added to the cells first, then ricin. Viability was assessed at 48 h. There was no difference in the ability of chRAC18 to protect any of the cells, regardless of the expression of FcR.

#### 2.2.5. Proteasome Inhibitor MG132 Affects neither Ricin Cytotoxicity nor Ab Protection

**Figure 9:**
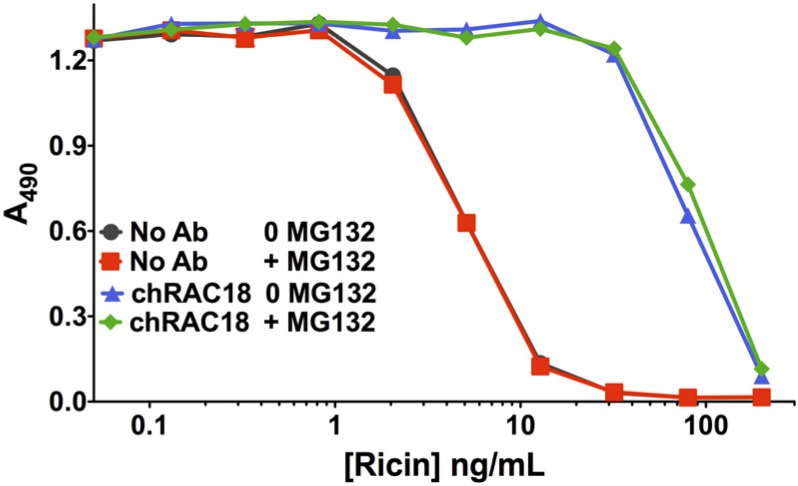
Inhibition of proteasome degradation does not affect ricin toxicity nor Ab neutralization. The drug MG132 inhibits proteasome degradation, a hallmark of TRIM21-mediated effects. An MTS assay was performed on H9 cells in the presence of 123 nM MG132 (370 nM MG132 completely suppresses MTS dye reduction) or in the absence of the drug. MG132 had no effect on toxicity or neutralization.

Inhibition of proteasome function has multiple consequences in cells, including inhibition of TRIM21, an internal Ig receptor that has been demonstrated to have an effect on Ab-mediated protection from intracellular microbes [17,18,19]. TRIM21 function is dependent upon proteasomal degradation [17]. Although it had been reported that inhibition of proteasomal degradation sensitized cells to ricin toxicity, a recent study showed that the duration of exposure of cells influenced effects of proteasomal inhibition, with short-term exposure (1–2 h) resulting in cytoprotection, and longer exposure (>6 h) having no effect on ricin toxicity [20]. A unique mechanism of action was demonstrated, emphasizing the pleotropic cellular effects of proteasome inhibition. We studied the effect of the proteasome inhibitor MG132 on ricin cytotoxicity and on Ab-mediated protection from ricin (Figure 9). MG132 was used at the maximum tolerable concentration and was present for the duration of the cytotoxicity assay. No effect was observed on either toxicity or neutralization, suggesting that prolonged proteasome inhibition has no effect on these processes. This confirms the data of Pietroni *et al.* [20] regarding ricin toxicity and extends them to include Ab-mediated neutralization of toxin.

## 3. Experimental Section

### 3.1. Antibodies, Cells and Reagents

Of a panel of 40 murine mAbs to ricin A or B chain, RAC18 bound ricin with highest avidity and was the most protective mAb, both *in vitro* and *in vivo* [3]. Hybridoma RAC18 was grown in tissue culture and purified on protein G agarose. A mouse-V, human-C chimeric RAC18 was produced in tissue culture expressed from recombinant plasmids [4]. Fab fragments of muRAC18 were prepared by digestion with immobilized papain and removal of Fc by protein G chromatography, as described [21]. RAC18 F(ab’)_2_ were prepared by pepsin digestion. Horse anti-ricin pAb was raised by hyperimmunization using a nontoxic ricin A and B chain construct, with the native linker between the A and B chains replaced by one that is not protease cleavable [5]. Fab/F(ab’)_2_ fragments were purified biochemically. IgG was purified by protein G chromatography. The concentration of anti-ricin antibody in these polyclonal preparations was determined by Biacore as shown in supplemental Figure S1 [22]. The concentration shown in the figures is the concentration of ricin-specific Ab.

H9 is a CD4+ human T-cell lymphoma tissue culture cell line [23], and was the gift of Marvin Reitz, then at NCI. TZM-bl are derived from HeLa transfected to express CD4, CCR5, and Tat-driven reporter genes, and were obtained from the NIH AIDS Reagent Program, Rockville MD [24,25]. We acknowledge the family of Henrietta Lacks for the donation of the original HeLa cells. TZM-bl cells transfected and stably expressing FcRγI, FcRγIIa, FcRγIIB, and FcRγIIIa were also obtained from the AIDS Reagent Program [12]. Cells were maintained in a humidified 5% CO_2_ atmosphere at 37° in RPMI 1640 medium with 10% fetal calf serum.

Goat anti-human, anti-horse, or anti-mouse IgG (heavy + light chains) or kappa chain was conjugated to either alkaline phosphatase (AP), horse radish peroxidase, or fluorescein isothiocyanate (FITC, Life Technologies, Inc, Grand Island, NY, USA). MAbs to human CD16, CD32, and CD64 were obtained from eBioscience. Ricin and RAC18 were conjugated to Alexa 488, 546, and/or 594 (Life Technologies) as described [4]. Brefeldin A BODIPY 558/568 (BFA-Bodipy), Lysotracker Blue DND-22 (LTB), and Hoechst 33258 were also purchased from Life Sciences. Ricin was obtained from Vector Labs (Burlingame, CA, USA). The proteasome inhibitor MG132 was obtained from Sigma Aldrich.

### 3.2. Enzyme Linked ImmunoSorbent Assay (ELISA)

ELISA plates were coated with ricin, 0.5 µg/mL, and blocked with blotto (10% non-fat dry milk/0.01% Tween 20/PBS) as described elsewhere [3]. Abs and fragments were diluted in blotto and incubated overnight in the coated wells at 4°. Wells were washed 6× PBS containing 0.01% Tween 20. Binding was detected with enzyme-conjugated anti-mouse kappa chain or anti-horse IgG (H + L). Plates underwent incubation for 4 h at room temperature before a washing step and addition of substrate. Absorbance was read on a microplate reader (BioTek 320, Winooski, VT, USA).

### 3.3. Neutralization of Ricin Cytotoxicity

Cell viability was measured by MTS dye reduction assay at 48 h. Ab-mediated neutralization of ricin was performed as detailed elsewhere [3]. Ricin and Ab were mixed in triplicate in 96-well, flat bottom tissue culture plates. Cells were added to a final volume of 200 µL. Cells were incubated at 37° in a humidified 5% CO_2_ atmosphere. Two days later, 30 µL of MTS/PMS dye (Promega, Madison, WI, USA) was added to each well. A_490_ was read hourly on a microplate reader. Percent viability was calculated as: [(A_test_ − A_no cells_)/(A_No ricin_ − A_no cells_)] × 100.

### 3.4. In Vivo Protection from Ricin Toxicity

Outbred NIH Swiss/Webster female mice 4–6 weeks of age were obtained from Charles River Laboratories (Wilmington, MA, USA). All experiments were done in compliance with federal laws and regulations and have been approved by the IACUC at The Research Institute for Children (OLAW assurance A-4336-01), following protocols described elsewhere [3,11]. Ricin (20 ug/kg) was administered via intraperitoneal injection. Antibody was administered by the same route four hrs later. Mice were examined and weighed twice daily. Blood sugar was measured 18 and 24 h post ricin. Mice were euthanized by an animal handler blinded to the experimental groups, according to pre-established criteria that defined the onset of pain, suffering, or clinical syndrome. All mice were sacrificed 7 days after ricin injection. Comparison of survival curves was by Mantel-Cox log rank test, computed using GraphPad Prism Software (version 5).

### 3.5. Flow Cytometry

Binding of Abs to cells was measured by flow cytometry, using both direct and indirect immunofluorescence. Cells were incubated sequentially with primary and secondary Abs in PBS/1% bovine serum albumin/0.01% sodium azide (PBA). After each incubation, cells were washed 3× with PBA. Ten thousand cells underwent analysis using an LSR II flow cytometer (BD Biosciences, San Jose, CA, USA) and analyzed with FlowJo software (Treestar, Ashland, OR, USA). Histograms displayed are based on cells gated first for SSC and FSC.

### 3.6. Live Cell Microscopy

Qualitative and quantitative confocal microscopy was performed on live cells using an inverted Zeiss LSM 510 microscope, with a heated stage and 63× 1.4 NA oil-immersion heated objective. Zeiss LSM and Axiovision software were used to obtain and analyze images. Full details of the experimental protocols, mathematical analyses, as well as appropriate controls, are provided elsewhere [4]. One day prior to imaging, 10^4^ cells were seeded into 35 mm culture dishes with a glass bottom of 0.17 mm thickness (MatTek, Ashland, MA, USA). Cells were cultured in 5% CO_2_ at 37° in RPMI 1640 containing no phenol red and 10% FCS. The following day, cells were placed into RPMI 1640, 1% bovine serum albumin (BSA), and 10 mM Hepes, and then transferred to the microscope stage for imaging. Experimental details for each study are provided in the figure captions. The brightness and contrast of confocal images shown in this manuscript have been adjusted to enhance visualization. Quantitative analyses were performed on the raw data.

## 4. Conclusions

In this report, we study the role of the Fc region of neutralizing Abs in protection from the toxic effects of ricin administration. We used both mAbs and Fabs, which differed in several ways. The mAbs were A chain specific, whereas the pAbs reacted with both A and B chains. The pAbs neutralized toxin *in vitro* at 10-fold lower concentrations than did the mAbs, but similar *in vivo* protection from ricin challenge was observed for both mAb and pAb. In comparing the function of intact Ig *vs.* Fab preparations of these two different sets of anti-ricin Abs, we found similar, and thus generalizable, results. *In vitro* functions (binding, neutralization) were unaffected by cleavage into fragments, although Fabs had lower avidity than F(ab’)_2_, likely due to decreased valence. However, *in vivo* there was a marked difference in protection, with intact Ig outperforming Fab and F(ab’)_2_. Moreover when tested in mice, murine mAb RAC18 outperformed its chimeric counterpart carrying human C regions, highlighting the importance of a homologous Fc.

We next studied the role of FcRγ on protection of cells from ricin toxicity because: (1) our *in vivo* observations showed the importance of the Fc region; (2) a study which used FcRγ knockout mice to demonstrate the requirement for FcRs in protection toxin [10]; and (3) our previous demonstration of intracellular neutralization of ricin toxin [4] led us to ask whether greater protection may be obtained if Ab has been imported into the cell by FcR, and is present prior to the internalization of ricin toxin. A panel of HeLa-derived cells carrying no FcR, human FcRγI, FcRγIIa, FcRγIIb, or FcRγIIIa was used to perform these studies. ChRAC18 (human IgG1) bound well to FcRγI and moderately to FcRγIII. Ab imported into cells by FcR-mediated endocytosis colocalized with ricin late during internalization and trafficking, most likely in the ER and Golgi. However, imported mAb did not offer the cell any protective advantage. When cells were exposed to ricin and mAb simultaneously, those carrying FcRγI imported greater amounts of ricin into the cells when mAb was present than when there was no mAb, whereas mAb had no effect on ricin internalization in FcR− cells. The presence or absence of FcR had no influence on mAb-mediated protection.

Overall, our findings indicate that neither the Fc region of the Ab nor the presence of FcR on the target cell has an influence on Ab-mediated protection of individual cells. However, *in vivo*, the presence of Fc enhances protective efficacy. We cannot say whether this effect as mediated by FcR, as suggested by others [10], by the prolonged plasma half-life of intact IgG *vs.* Fab, decreased avidity of Fab fragments, or by some other completely different Fc-mediated mechanism. When using xenogeneic antibodies for passive immune therapy, Fab/F(ab’)_2_ preparations may be less likely to provoke an immune response and induce serum sickness. However, this advantage should be weighed against the potential loss of *in vivo* protective activity, compared to intact Ig.

## Supplementary Files

Supplementary File 1 Supplementary Information (PDF, 138 KB)
