# Supplementary material for: Role of Fc in Antibody-Mediated Protection from Ricin Toxin
Source: Toxins (Basel). 2014 May 7;6(5):1512–25. doi: 10.3390/toxins6051512 (PMC4052250; doi:10.3390/toxins6051512)
Supplement: Supplementary File 1 — Supplementary Information (PDF, 138 KB) [file toxins-06-01512-s001.pdf]

Supplementary Information

Figure S1. Biacore analysis to determine the concentration of anti-ricin Ab in polyclonal Fab/F(ab')$_2$ preparations. Serial dilutions of Ab or fragments (30–250 nM, based on total protein concentration) were captured on a ricin-coated sensor chip under mass transport limited conditions (flow rate 5 µL/min) or non-mass transport limited (100 µL/min) conditions. Concentration of anti-ricin Ab or fragment was determined according to the following calculations [21].

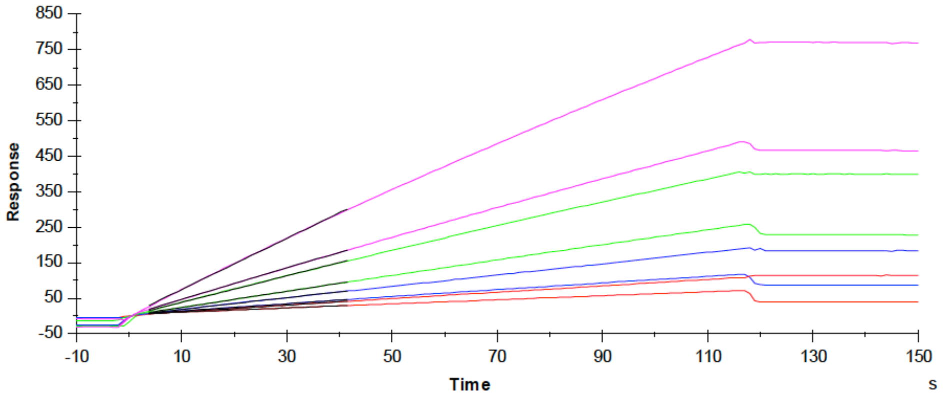

The concentration of the analyte [Abulk] is calculated by the following equation:

$$[A_{\mathbf{bulk}}] = \frac{\frac{1}{L_{m1}} - \frac{1}{L_{m2}}}{\mathrm{MW} \cdot G \cdot \left(\frac{1}{\frac{dR_1}{dt_1}} - \frac{1}{\frac{dR_2}{dt_2}} \right)}$$

L_m = mass transport coefficient

MW = 100,000g/mole for F(AB')$_2$

G = 1000RU ·mm^2/ng = 1x10^6 RU·m^2/g

dR/dt = linear slope (measured within the first ~36s of binding where k_d is negligible) of 2 curves

1. Mass transport limited (flow 5 µl/min) $\quad$ flw$_5$ $= 8.33\ x\ 10^{-11}\ \mathrm{m}^3/\mathrm{s}$
2. Non mass transport (flow 100 µl/min) $\quad$ flw$_{100}$ $= 1.67\ x\ 10^{-9}\ \mathrm{m}^3/\mathrm{s}$

L_m in m/s is calculated as follows:

$$L_m = \sqrt[3]{\frac{D^2 \cdot \mathrm{flw}}{\mathrm{h}^2 \cdot \mathrm{w} \cdot \mathrm{l}}}$$

h	height of the flow cell	= 1.8 x10^{-5} m
w	width of the flow cell	= 5 x 10^{-4} m
l	length of the flow cell	= 1.6 x 10^{-3} m

D the diffusion coefficient for F(AB')$_2$ at 37°C calculated as follows:

$$D = \frac{1.0x10^{-11} \cdot 324.3 \cdot MW^{-\left(\frac{1}{3}\right)} \cdot C_{t37}}{\frac{f}{fo} \cdot \frac{v}{vo}}$$

f/fo	friction factor	=1.2
v/vo	solution viscosity	=0.89
C_{t37}	conversion to 37°C	=1.06
